# Selective pulmonary lobe isolation with Arndt pediatric endobronchial blocker for an infant: A case report

**DOI:** 10.1097/MD.0000000000018262

**Published:** 2019-12-16

**Authors:** Chaomeng Wu, Xiao Liang, Bin Liu

**Affiliations:** Department of Anesthesiology, West China Hospital of Sichuan University, 37 Guoxue Alley, Chengdu, Sichuan, PR China.

**Keywords:** infants, pulmonary lobe isolation, thoracic surgery

## Abstract

**Introduction:**

Attaining lung isolation in the infant is a challenge for anesthesia care providers. Pulmonary lobe isolation is more challenging. We describe an approach to performing selective pulmonary lobe isolation using the pediatric endobronchial blocker in an infant in the absence of appropriate auxiliary guidance tool.

**Patient concerns:**

An 8-month-old and 9.5 kg male infant was admitted because of repeated cough with fever for 3 months and a large cyst of his right lung for 2 weeks. He had been living in a pastoral area with his parents.

**Diagnosis:**

Based on the chest computed tomography (CT) and his history about long-term residence in the pastoral area, this patient's diagnosis was considered as right middle lobe hydatid cyst.

**Interventions:**

Guided by a fiberoptic bronchoscope, a cuffed 4.0-mm inside diameter (ID) endotracheal tube was successfully placed into the right main bronchus of this infant. Then, pediatric 5-French (Fr) endobronchial blocker was placed into the right middle and lower lobes through the endotracheal tube without navigation of fiberoptic bronchoscope.

**Outcomes:**

Lobe isolation was successfully achieved for right middle lobectomy. This approach allows clinicians to perform lobe isolation in the absence of fiberoptic bronchoscope with very small outer diameter.

**Conclusion:**

This technique is relatively easy to use and less dependent on equipment with small outer diameter in the selective pulmonary lobe isolation in infants and small children.

## 1 Introduction

The increasing frequency of thoracic surgical procedures in infants demands appropriate anesthetic techniques for lung isolation. Isolating lung lobe instead of an entire lung is probably advantageous, especially in infant patients with pulmonary hydatid cyst, to effectively prevent section diffusion of operative lung lobe(s) while effectively maintaining intraoperative ventilation and oxygenation. However, it is challenging to attain lobe isolation for infants or small children due to their unique anatomy. With the limitation or lack of bronchial intubation devices available for infants or small children,^[1]^ the anesthesiologists have been attempting various methods to achieve the lung isolation or lobe isolation to strive more surgical treatment opportunities for these infants and small children.

## 2 Methods

In this report, we described a practical technique for successfully isolation of right pulmonary middle lobe in an infant for resection of a large pulmonary cyst in the absence of appropriate auxiliary guidance tool. Informed consent, which was approved by the West China Hospital Institutional Review Board, was obtained from the patient's parents prior to participation in the study.

## 3 Case report

### 3.1 Patient information

An 8-month-old and 9.5 kg male infant was treated in a local village clinic for repeated cough and fever 3 months ago. Due to poor results, he was transferred to a county hospital for chest Computed Tomography (CT) 2 weeks ago. A large cyst of his right lung was found. The cyst measured 3 × 4 × 4 cm^3^ and was confined to the middle lobe of the right lung. It was considered to be a hydatid cyst because the patient was from a pastoral area. Then, he was transferred to our hospital for the elective resection of a right middle lobe hydatid cyst.

### 3.2 Anesthesia process and surgical procedure

**Figure 1:**
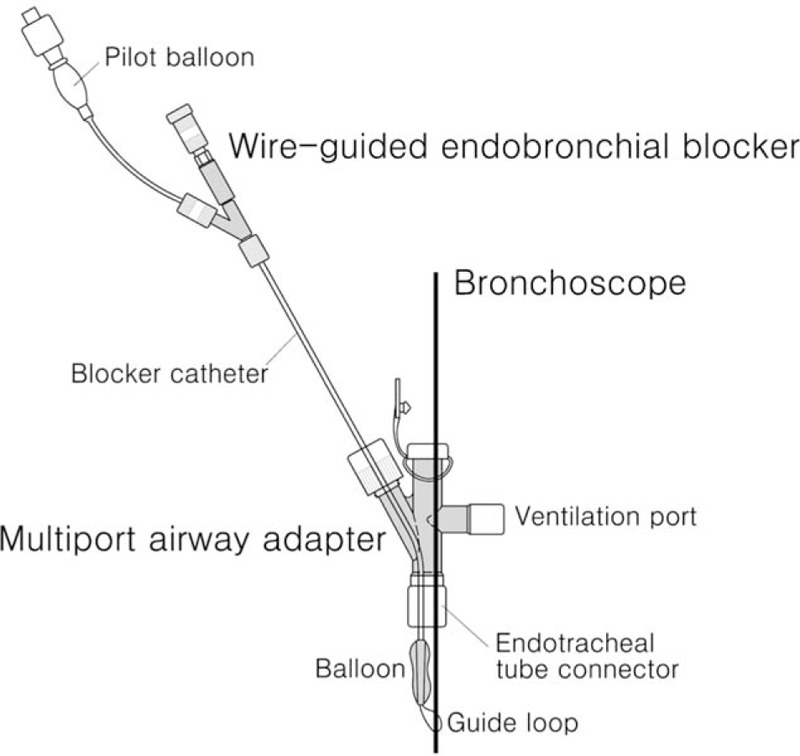
The fiberoptic bronchoscope and the pediatric wire-guided endobronchial blocker were operated through the multiport airway adapter, respectively. The lumen of 4.0-mm ID ETT is too small to allow both of them to go through simultaneously.

Standard monitoring was applied including electrocardiogram, non-invasive blood pressure (NBP), end tidal carbon dioxide (ETCO_2_), pulse oximetry (SpO_2_) and temperature. Patient was induced intravenously with 1 mg midazolam, 30 μg fentanyl, 1 mg vecuronium and 20 mg propofol. A standard cuffed 4.0-mm ID endotracheal tube (ETT) was successfully inserted into the trachea. Anesthesia was maintained with sevoflurane 3%. The fiberoptic bronchoscope [outside diameter (OD) 2.8 mm] and an Arndt pediatric endobronchial blocker (5-Fr catheter) were operated through the multiport airway adapter, respectively (Fig. 1).

**Figure 2:**
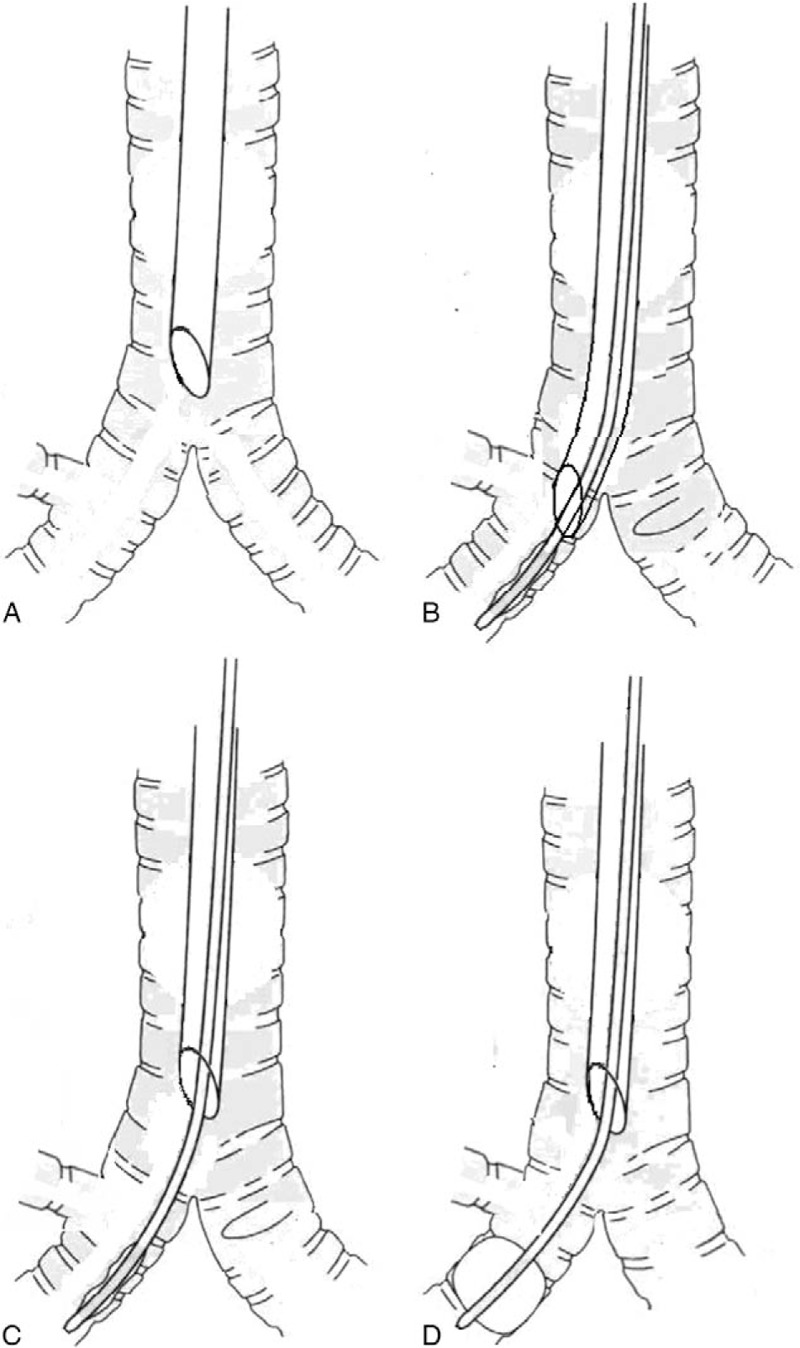
Placement of endobronchial blocker. A: A standard cuffed 4.0-mm ID ETT was successfully inserted into the trachea. And then the ETT was advanced into right bronchus with navigation of fiberoptic bronchoscope. B: The tip of ETT was placed distally to the right upper lobe bronchus opening. Then fiberoptic bronchoscope was removed, and endobronchial blocker was advanced with the entire balloon of the endobronchial blocker just beyond the tip of ETT per the scale of this blocker. C: The ETT was withdrawn to the level 10 mm above the carina. D: The tip of the endobronchial blocker remained in the right bronchus.

ETT was advanced into the right bronchus with navigation of fiberoptic bronchoscope (Fig. 2A). The tip of ETT was placed distally to the right upper lobe bronchus opening. Then, fiberoptic bronchoscope was removed and endobronchial blocker was advanced with the entire balloon of the endobronchial blocker just beyond the tip of ETT per the scale of this blocker (Fig. 2B). The endotracheal tube was withdrawn to the level 10 mm above the carina (Fig. 2C), and the tip of the endobronchial blocker remained in the right bronchus (Fig. 2D). The correct cuff position of the endobronchial blocker was identified by auscultation of bilateral lung fields with endobronchial blocker cuff inflation. When the breathing sound of the middle and lower lobes disappeared up on inflation of the endobronchial blocker cuff and that of the upper lobe could still be heard, the middle and lower lobes were isolated from the right upper lobe. The guide loop (nylon wire) of endobronchial blocker was removed, and the central lumen was used for suctioning and oxygen supply.

The correct lobe isolation was further confirmed by direct vision upon surgical exposure after placing the patient into the left lateral decubitus position. The right middle and lower lobes of the lung were well deflated. The right middle lobectomy was completed in 55 minutes uneventfully. SpO_2_ of this infant remained above 93% during lobes isolation with intermittent oxygen flow of 1.2 L/min via the lumen of endobronchial blocker. Upon completion of the lobectomy, the secretions were suctioned through the endobronchial blocker, and then the cuff was deflated. The right lower lobe was reinflated. At the end of the procedure, the infant was returned to the supine position and successfully extubated. His postoperative course was unremarkable, and he was discharged home on postoperative day 5.

## 4 Discussion

Thoracic surgeries often require lung isolation. The lobe isolation is also needed in many situations for correction of congenital lung abnormalities. It is either needed for identification of fissures between the lobes or preventing blood and secretions into non-operative lobe(s).^[2]^ In this report, the infant underwent resection of a pulmonary cyst, a procedure that commonly resulted in the release of copious mucoid, purulent and/or bloody secretions from the cyst to the ipsilateral main-stem bronchus. By isolating the patient's operative lobes with a bronchial blocker and suctioning the secretions, we were successful in avoiding this complication.

There are a variety of techniques available for achieving the lung isolation in the adults and large children, but the options for the infants and small children are limited.^[1,3–6]^ A fibreoptically-directed wire-guided 5Fr endobronchial blocker had been developed for lung isolation in infant and small children.^[7]^ When using the coaxial technique, the Arndt endobronchial blocker worked well.^[8]^ Samuel Wald reported 24 patients (aged 2–16 year) received single lung ventilation using the pediatric endobronchial blocker with the same approach as it was used in adults.^[9]^ Although very small fiberoptic bronchoscope with OD 2.0 mm or 2.2 mm can be used for positioning the cuff of endobronchial blocker in target depth for infants and small children,^[9–11]^ the issue with that approach is unavailability of such a small size fiberoptic bronchoscope in many institutes like ours.

This is the first presentation of the using 5-Fr pediatric Arndt endobronchial blocker in an infant of less than 10 kg to successfully achieve lobe isolation in the absence of more appropriate auxiliary tool such as fiberoptic bronchoscope with OD less than 2.8 mm. From this report, we found that the approach should be readily applicable if the lung isolation was needed in infants or small children. Because the lumen of 4.0-mm ETT is too small to allow both endobronchial blocker and fiberoptic bronchoscope to go through simultaneously, we inserted the ETT with navigation of the fiberoptic bronchoscope into the target position. Then we removed the fiberoptic bronchoscope and used the ETT to guide insertion of endobronchial blocker to right bronchus. The target depth of endobronchial blocker insertion was adjusted with the scale on the ETT and endobronchial blocker. Indeed, the insertion of endobronchial blocker was achieved blind. Initial identification of correct positioning the blocker cuff was obtained with auscultation and final confirmation was by direct visualization of the target lobe deflation and the rest lobes’ adequate inflation. The correct insertion of the cuff and lobe isolation was not only achieved without much difficulty but also well maintained during the entire surgery.

With the technique we described above, clinicians can position the endobronchial blocker using the ETT pre-positioned with navigation of fiberoptic bronchoscope, and the insertion and positioning of the endobronchial blocker is obtained blindly. We believe that this technique is particularly useful in institutes like ours in which the fiberoptic bronchoscope of very small OD (less than 2.8 mm) is unavailable.

There are also a few other techniques previously described lung isolation for infant and small children. John L Bastien demonstrated extraluminal use of the pediatric endobronchial blocker with 2.2 mm fiberoptic bronchoscope in an infant.^[10]^ Christoph Schmidt made 3 distinctive modifications of the standard procedure in a newborn using the pediatric endobronchial blocker with 2.0 mm fiberoptic bronchoscope.^[11]^ Comparing with other techniques, we believe that our approach has following advantages:

1) there is no limitation of size of the endotracheal tube;
2) minimizing hypoxia due to reduction in shunt;
3) oxygen can be administered to the operative lung if the infant and small children are experiencing oxygen desaturation;
4) selective pulmonary lobe isolation provides clear borderline of pulmonary lobes; and
5) suction can be applied to the operative lobes to promote lung collapse and remove secretions.

The other very important step is to estimate the distance between the target position of the endobronchial blocker and the carina of trachea by radiographic imaging such as chest CT or fiberoptic bronchoscopy, so that the endobronchial blocker can be accurately placed with the scale on the ETT and endobronchial blocker as soon as possible, reducing the risk of airway trauma caused by repeated adjustments of the position of endobronchial blocker's balloon.

## 5 Conclusion

We have developed a useful method to attain selective pulmonary lobe isolation in infants using endobronchial blocker. This technique is readily applicable and less dependent on equipment with small outer diameter in the selective pulmonary lobe isolation in infants and small children.

## Author contributions

**Conceptualization:** Bin Liu.

**Investigation:** Chaomeng Wu.

**Resources:** Xiao Liang.

**Writing – original draft:** Chaomeng Wu.

**Writing – review & editing:** Xiao Liang.
